# Early prediction of noninvasive ventilation failure after extubation: development and validation of a machine-learning model

**DOI:** 10.1186/s12890-022-02096-7

**Published:** 2022-08-08

**Authors:** Huan Wang, Qin-Yu Zhao, Jing-Chao Luo, Kai Liu, Shen-Ji Yu, Jie-Fei Ma, Ming-Hao Luo, Guang-Wei Hao, Ying Su, Yi-Jie Zhang, Guo-Wei Tu, Zhe Luo

**Affiliations:** 1 Department of Critical Care Medicine, Zhongshan Hospital, Fudan University, Shanghai, China; 2 College of Engineering and Computer Science, Australian National University, Canberra, ACT Australia; 3 Shanghai Medical College, Fudan University, Shanghai, China; 4 Department of Critical Care Medicine, Xiamen Branch, Zhongshan Hospital, Fudan University, Xiamen, China; 5 Shanghai Key Lab of Pulmonary Inflammation and Injury, Shanghai, China

**Keywords:** Non-invasive mechanical ventilation failure, Recursive feature elimination, Hyperparameter optimization, Categorical Boosting, Prospective validation

## Abstract

**Background:**

Noninvasive ventilation (NIV) has been widely used in critically ill patients after extubation. However, NIV failure is associated with poor outcomes. This study aimed to determine early predictors of NIV failure and to construct an accurate machine-learning model to identify patients at risks of NIV failure after extubation in intensive care units (ICUs).

**Methods:**

Patients who underwent NIV after extubation in the eICU Collaborative Research Database (eICU-CRD) were included. NIV failure was defined as need for invasive ventilatory support (reintubation or tracheotomy) or death after NIV initiation. A total of 93 clinical and laboratory variables were assessed, and the recursive feature elimination algorithm was used to select key features. Hyperparameter optimization was conducted with an automated machine-learning toolkit called Neural Network Intelligence. A machine-learning model called Categorical Boosting (CatBoost) was developed and compared with nine other models. The model was then prospectively validated among patients enrolled in the Cardiac Surgical ICU of Zhongshan Hospital, Fudan University.

**Results:**

Of 929 patients included in the eICU-CRD cohort, 248 (26.7%) had NIV failure. The time from extubation to NIV, age, Glasgow Coma Scale (GCS) score, heart rate, respiratory rate, mean blood pressure (MBP), saturation of pulse oxygen (SpO_2_), temperature, glucose, pH, pressure of oxygen in blood (PaO_2_), urine output, input volume, ventilation duration, and mean airway pressure were selected. After hyperparameter optimization, our model showed the greatest accuracy in predicting NIV failure (AUROC: 0.872 [95% CI 0.82–0.92]) among all predictive methods in an internal validation. In the prospective validation cohort, our model was also superior (AUROC: 0.846 [95% CI 0.80–0.89]). The sensitivity and specificity in the prediction group is 89% and 75%, while in the validation group they are 90% and 70%. MV duration and respiratory rate were the most important features. Additionally, we developed a web-based tool to help clinicians use our model.

**Conclusions:**

This study developed and prospectively validated the CatBoost model, which can be used to identify patients who are at risk of NIV failure. Thus, those patients might benefit from early triage and more intensive monitoring.

*Trial registration*: NCT03704324. Registered 1 September 2018, https://register.clinicaltrials.gov.

**Supplementary Information:**

The online version contains supplementary material available at 10.1186/s12890-022-02096-7.

## Background

Noninvasive ventilation (NIV) has been widely used and is currently deemed a promising therapy [1]. Its main benefits include: (1) providing oxygen and pressure support for patients with hypoxia and respiratory failure to avoid intubation; (2) facilitating ventilator weaning, when used sequentially or early after extubation. Compared with conventional oxygen therapy, NIV can offer more support and reduce the need for endotracheal intubation. In contrast to invasive mechanical ventilation, NIV causes fewer relevant complications, such as pneumonia or ventilator-induced lung injury [2].

However, a substantial proportion of patients (5–60%) experience NIV failure, owing to miscellaneous factors, including acute respiratory failure or congestive heart failure and so on. A considerable number of people may receive insufficient support or may experience NIV intolerance. Other situations in which patient condition may deteriorate without obvious signs may also lead to NIV failure. It was reported that low pH, low Glasgow coma scale score, low oxygenation, high heart rate, high respiratory rate, and high tidal volume were associated with NIV failure [3–7]. More importantly, NIV failure is strongly linked to poor outcomes: the mortality rate of patients with NIV failure and reintubation is markedly higher than that of patients with successful ventilation [8]. In addition, patients with delayed reintubation have a higher mortality rate than patients reintubated earlier [9, 10].

Accordingly, the assessment of NIV efficacy and timely subsequent treatment decisions appear to be particularly crucial. Previous predictive studies have used not only single variables, such as the rapid shallow breathing index (RSBI), but also comprehensive parameters to predict NIV failure. For instance, Duan et al. have used a combination of variables including heart rate, acidosis, consciousness, oxygenation, and respiratory rate to develop a risk-scoring system for early prediction of NIV failure among patients with COPD [10]. Moreover, Liu et al. have developed a simple nomogram for predicting failure of noninvasive strategies in adults with COVID-19 [10, 11]. In the present study, considering post-extubation respiratory failure and congestive heart failure, etc. we enrolled all critical patients undergoing NIV after extubation, without limiting the types of primary illness leading to intubation. Moreover, some prior studies have explored the ability of machine-learning models to accurately predict extubation failure in recent years showing remarkable accuracy [12, 13].

Although certain parameters have been demonstrated to predict NIV failure after extubation, reports of using multiple variables with machine learning based on a large database remain scant. To help clinical decision, the present study aimed at developing and validating a feasible and reliable machine learning model to predict NIV failure in patients receiving NIV after extubation.

We present the following article in accordance with the TRIPOD reporting checklist.^1^

## Methods

### Source of data

The eICU-CRD database, a multicenter database comprising de-identified health data associated with more than 200,000 admissions to ICUs across the United States between 2014 and 2015, was used to develop the predictive model. One author (QZ) obtained access to the database and was responsible for data extraction. For external validation, a post cardiac surgical NIV dataset was extracted form a prospective cohort in our setting (PREDICt study, NCT 03704324). The study was conducted in accordance with the Declarations ration of Helsinki (as revised in 2013). The study was approved by institutional committee board of Zhongshan Hospital, Fudan University (NO. B2018-071) and informed consent was taken from all the patients. This cohort was approved by the relevant institutional ethics committee (approval No. B2018-071). The study is reported according to the recommendations of the Transparent Reporting of a multivariable prediction model for Individual Prognosis or Diagnosis (TRIPOD) statement [14].

### Selection of participants

**Fig. 1:**
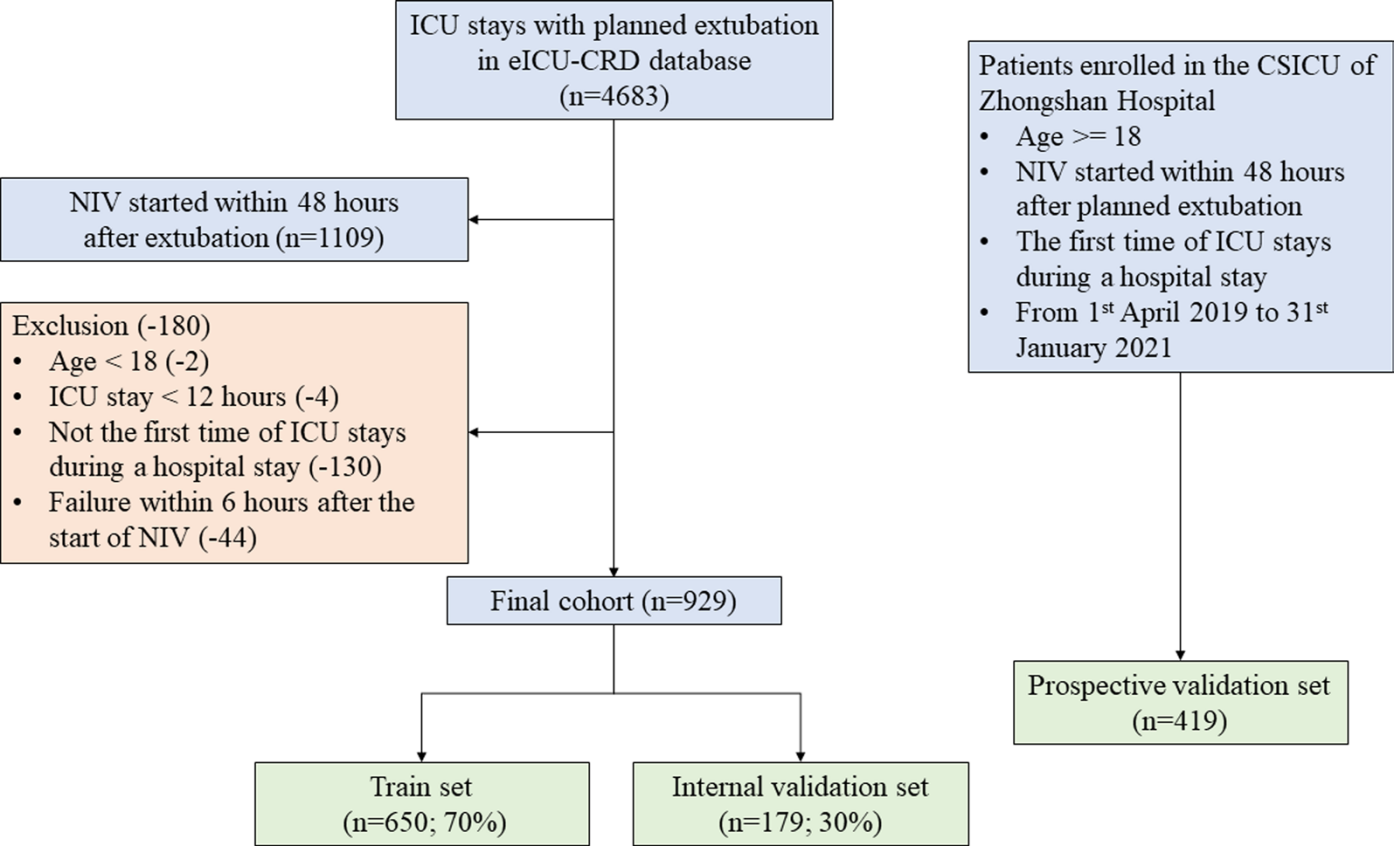
Flow chart of patient selection. eICU-CRD, eICU Collaborative Research Database; ICU, intensive care unit

In the eICU-CRD cohort, patients who underwent extubation during ICU stays were included (Fig. 1). The exclusion criteria were: (i) age < 18 years, (ii) unplanned extubation, (iii) ICU stay < 12 h, or (iv) reintubation or death within 6 h after the initiation of NIV. In the PREDICt cohort, all patients who did not meet the above exclusion criteria from April 2019 to January 2021 were prospectively enrolled. Informed consent was obtained from patients’ legally authorized representatives after admission to the ICU.

No patients were excluded because of missing values; instead, all eligible patients in eICU-CRD were included to maximize the statistical power of the predictive model.

### Data collection and outcome definition

In the eICU-CRD cohort, clinical and laboratory variables were extracted within 6 h after the initiation of NIV (Additional file 1: Table S1), including patient characteristics (age, sex, and ethnicity), laboratory data (arterial blood gas, full blood count, liver function, and renal function), and vital signs (respiratory rate, blood pressure, heart rate, SpO_2_, and temperature). For some variables with multiple measurements, such as mean airway pressure, heart rate, etc., average values were assessed. The average amount of input (crystalloid bolus or colloid bolus) and that of output (urine output) were calculated within 6 h after the initiation of NIV, and were then normalized to patient weight. Glasgow Coma Scale assesses the degree of coma in three areas: eye opening response, verbal response and body movement. And the sum of the three areas is the coma index, which was extracted from nursing records. The time from extubation to NIV and ventilation duration is time interval which was also extracted from records. Peripheral oxygen saturation (SpO_2_), assessed by bedside vital signs monitor, is an estimation of the oxygen saturation level usually measured with a pulse oximeter device. Pressure of oxygen in blood (PaO_2_) means the tension created by the oxygen physically dissolving in the blood, which is assessed during blood gas analysis. Mean airway pressure (Pmean), showed on the ventilator, determined by the peak inspiratory pressure (PIP), PEEP, and the inspiratory to expiratory time ratio [15]. The mean airway pressure was measured for NIV within 6 h of initiation. On most ventilators, airway pressure is measured directly by a pressure sensor and displayed on the ventilator screen. Generally, Pmean closely correlates with mean alveolar pressure and may represent the stresses applied to the lung parenchyma with ventilation [16]. Furthermore, comorbidities were also assessed on the basis of the recorded International Classification of Diseases (ICD)-9 and ICD-10 codes [17], and the Charlson Comorbidity Index was calculated [18]. In addition, we extracted data on medications such as heparin, antibiotics, and vasopressors, as well as continuous renal replacement therapy. Finally, the hospital mortality, reintubation, and initiation of NIV after extubation were also assessed.

Generally, the definition of NIV failure was a need for endotracheal intubation or death [19]. The primary outcome of this study was the need for invasive ventilatory support (reintubation or tracheotomy) or death after NIV initiation [20].

### Statistical analysis

Baseline characteristics were compared between the NIV success group and the failure group in the eICU-CRD and PREDICt cohorts. For continuous variables, values are presented as the means (standard deviations) (if normal) or medians [interquartile ranges] (if non-normal), and comparisons were made with the Student’s *t*-test or rank-sum test, as appropriate. For categorical variables, values are presented as total numbers [percentages], and chi-square tests or Fisher's exact tests were used, as appropriate, to examine differences between groups.

**Fig. 2:**
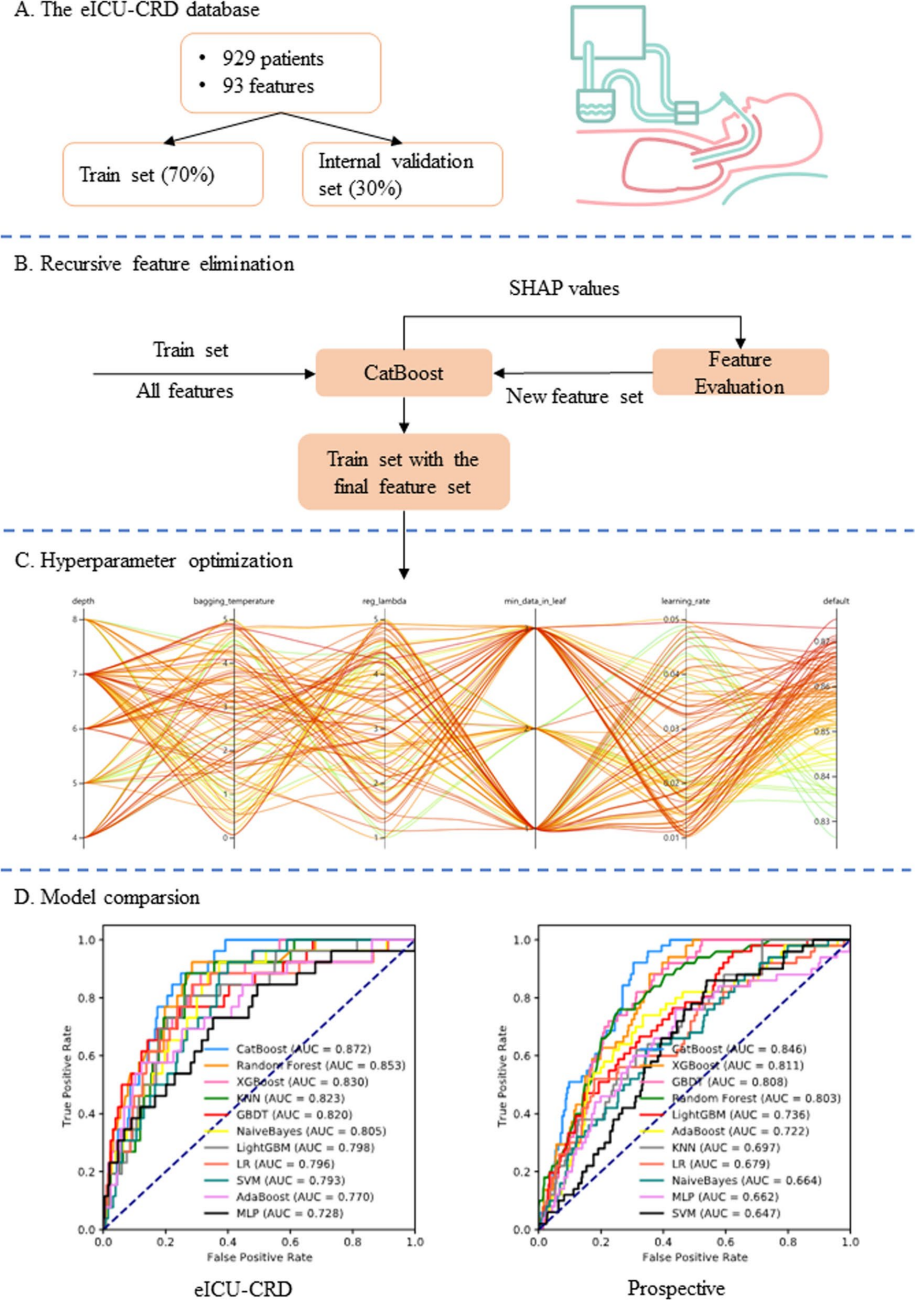
Schematic illustration of the study design. **A** Patients with NIV initiated within 48 h after extubation in the eICU Collaborative Research Database were included in the study, and 93 variables were extracted. The dataset was divided into a training set (70%) and internal validation set (30%). **B** The recursive feature elimination algorithms were performed on the training set, and key features were selected. **C** Hyperparameters was optimized by using an automated machine learning toolkit on the training set. **D** The developed CatBoost model outperformed other models in both the internal validation and prospective validation sets

An advanced machine-learning model called CatBoost was developed using the catboost Python package (version 0.24). As shown in Fig. 1, the eICU-CRD dataset was first randomly split into a training set (70%) and internal validation set (30%). Of note, categorical variables or missing values were not processed because the CatBoost algorithm was able to handle them automatically. Second, the recursive feature elimination (RFE) algorithm based on SHapley Additive exPlanations (SHAP) values was used to screen out key features, as shown in Fig. 2B. In short, a full CatBoost model was developed on the basis of the training set with all available variables to predict NIV failure. Second-order variables calculated on the basis of other variables, such as RSBI, Sequential Organ Failure Assessment (SOFA) and Simplified Acute Physiology Score (SAPS)-II, were manually excluded. The effects of the remaining features on prediction scores were then measured with the functions of the SHAP Python package (version 0.32.1), which assessed the importance of each feature through a game-theory approach [21]. The feature with the smallest effect on the prediction was eliminated in each loop, and a new CatBoost model was recursively fitted on the basis of smaller feature sets until a significant decrease in model performance was observed [22]. Finally, the key features that had the greatest importance and could be easily collected in clinical settings were selected.

To further improve the model performance, we conducted hyperparameter tuning by using an automated machine learning toolkit called Neural Network Intelligence designed by Microsoft Research. We chose the Tree-structured Parzen Estimator, a sequential model-based optimization algorithm, as the tuning algorithm, which sequentially constructs models to approximate the performance of hyperparameters according to historical measurements, then subsequently chooses new hyperparameters to test on the basis of this model (Bergstra et al., 2011). The hyperparameter search domain is summarized in Additional file 2: Table S2. A total of 100 trails were examined, and the parameters with the greatest area under the receiver operating characteristic (AUROC) were saved. A compact CatBoost model using the saved parameters was then trained on the selected features, then validated in the validation sets.

AUROCs were also calculated to compare our model with other predictive factors commonly used in ICUs, such as RSBI and the ratio of pulse oximetry/fraction of inspired oxygen to respiratory rate. Additionally, ten different models were derived on the training set and compared with our CatBoost model, including K-Nearest Neighbor (KNN), AdaBoost, Multi-Layer Perceptron (MLP), Support Vector Machine (SVM), Logistic Regression (LR), NaiveBayes, Gradient Boosting Decision Tree (GBDT), random forest, LightGBM, and eXtremely Gradient Boosting (XGBoost) [23]. Of note most of these models cannot analyze data with missing values, and therefore datasets were imputed with multiple imputation [24]. Furthermore, categorical variables were converted to one-hot encoding, and data were centered on zero and scaled before training of the KNN, MLP, SVM, LR, and NaiveBayes models. These nine models and our CatBoost model were compared in both the internal and prospective validation sets (shown in Fig. 2D).

All statistical analyses in the present study were performed with Python (version 3.7.6); *P* < 0.05 was considered statistically significant.^2^

## Results

### Baseline characteristics

A flow chart of patient selection is shown in Fig. 1. In this study, 929 and 419 patients who experienced NIV within 48 h after extubation were eventually included in the eICU-CRD and PREDICt cohorts, respectively. The eICU-CRD dataset was divided into a training set (n = 650; 70%) and internal validation set (n = 179; 30%). During the enrollment period, 419 patients were eligible for inclusion in the external validation cohort, and no patients were excluded because of missing data.

The comparison of baseline characteristics between the NIV success and failure groups in the eICU-CRD and PREDICt cohorts is summarized in Table 1. Variables with missing data are shown in the appendix. The ratio of patients who experienced NIV failure was 26.7% (n = 248) in the eICU-CRD group and 20.5% (n = 86) in the PREDICt cohort. In the eICU-CRD cohort, the NIV failure group had higher heart rate, respiratory rate, glucose, urine output, input, mean airway pressure and longer mechanical ventilation duration (*p* < 0.05). A significantly lower GCS score and PaO_2_ were observed in the failure group. Besides, time from extubation to NIV, GCS score, SpO_2_, PaO_2_, mechanical ventilation duration, and mean airway pressure significantly differed between the NIV success and failure groups in the PREDICt cohort. Higher mean airway pressure, and longer mechanical ventilation duration were observed in the NIV failure group than in the success group.

**Table 1:** Baseline characteristics of the eICU-CRD and PREDICt cohorts

	eICU-CRD cohort					PREDICt cohort				
	Missing	Overall	NIV success	NIV failure	*p* value	Missing	Overall	NIV success	NIV failure	*p* value
n		929	681	248			419	333	86	
Ext. To NIV ≤ 24 h, n (%) h	0	876 (94.3)	647 (95.0)	229 (92.3)	0.164	0	348 (83.1)	289 (86.8)	59 (68.6)	< 0.001
Age, mean (SD) years	0	62 (15)	62 (15)	61 (15)	0.374	1	61 (14)	61 (14)	63 (12)	0.120
GCS, median [Q1, Q3]	263	14 [12, 15]	15 [13, 15]	11 [9, 15]	< 0.001	0	15 [13, 15]	15 [13, 15]	14 [13, 15]	< 0.001
Heart Rate, mean (SD) beats per min	3	88 (15)	87 (15)	91 (16)	0.001	31	95 (20)	95 (20)	97 (18)	0.433
Respiratory rate, mean (SD) beats per min	14	21 (5)	20 (5)	22 (6)	< 0.001	31	21 (7)	21 (7)	22 (6)	0.370
MBP, mean (SD) beats per min	15	82 (14)	82 (14)	82 (13)	0.689	37	81 (12)	81 (12)	82 (11)	0.409
SpO2, median [Q1, Q3]	64	97 [95, 98]	97 [95, 98]	97 [95, 98]	0.312	47	99 [96, 100]	99 [97, 100]	98 [96, 100]	0.048
Temperature, mean (SD) ℃	39	37.2 (0.6)	37.2 (0.6)	37.2 (0.7)	0.574	8	37.3 (0.7)	37.3 (0.7)	37.2 (0.7)	0.177
Glucose, mean (SD) mg/dl	132	131 (39)	129 (35)	138 (49)	0.010	59	160 (39)	159 (38)	165 (42)	0.271
pH, mean (SD)	567	7.38 (0.06)	7.38 (0.06)	7.36 (0.08)	0.058	51	7.44 (0.09)	7.44 (0.08)	7.42 (0.13)	0.263
PaO_2_, median [Q1, Q3] mmHg	568	89 [74, 113]	91 [75, 113]	84 [67, 106]	0.039	52	103 [81, 137]	99 [76, 132]	79 [62, 107]	< 0.001
Urine Output, median [Q1, Q3] mL/kg/h	3	0.6 [0.0, 1.2]	0.6 [0.0, 1.2]	0.7 [0.2, 1.4]	0.039	54	1.8 [1.4, 2.3]	1.8 [1.4, 2.4]	1.8 [1.5, 2.2]	0.823
Input, median [Q1, Q3] mL/kg/h	3	0.1 [0.0, 0.6]	0.0 [0.0, 0.6]	0.2 [0.0, 0.9]	< 0.001	62	1.3 [1.1, 1.7]	1.3 [1.1, 1.8]	1.4 [1.1, 1.7]	0.521
Mechanical Ventilation Duration, median [Q1, Q3] h	2	34.5 [12.4, 96.0]	22.7 [8.1, 51.2]	121.0 [55.4, 227.6]	< 0.001	10	28.0 [18.0, 62.0]	25.0 [18.0, 47.0]	61.5 [18.2, 94.5]	< 0.001
Mean Airway Pressure, mean (SD) cmH_2_O	555	10.2 (7.3)	8.5 (6.9)	12.9 (7.0)	< 0.001	0	9.8 (1.6)	9.5 (1.1)	11.1 (2.2)	< 0.001
Failure Type										
Tracheotomy	–	–	–	45 (18.1)	–	–	–	–	51 (59.3)	–
Reintubation	–	–	–	210 (84.7)	–	–	–	–	75 (87.2)	–
Mortality	–	–	–	80 (32.4)	–	–	–	–	31 (36.0)	–

Values are presented as the mean (SD) if not otherwise specified

eICU-CRD, eICU Collaborative Research Database; ZS, Zhongshan; NIV, noninvasive ventilation; GCS, Glasgow Coma Scale Score; MBP, mean blood pressure; SpO_2_, saturation of peripheral oxygen; pH, potential of hydrogen; PaO_2_, arterial partial pressure of oxygen; NIV, noninvasive ventilation

Moreover, in the PREDICt cohort, a significant difference was observed in the duration from extubation to NIV initiation (≤ 24 h/ > 24 h) (*p* < 0.05). Reintubation rate was 22.6% (n = 210) in the eICU-CRD group while it was 17.9% (n = 75) in the PREDICt cohort. Furthermore, 80 patients in the eICU-CRD group died during hospitalization, accounting for 32.4% of the patients with NIV failure (n = 248). In the PREDICt cohort, the hospital mortality was 36% (n = 31).

### Development of the CatBoost model

**Fig. 3:**
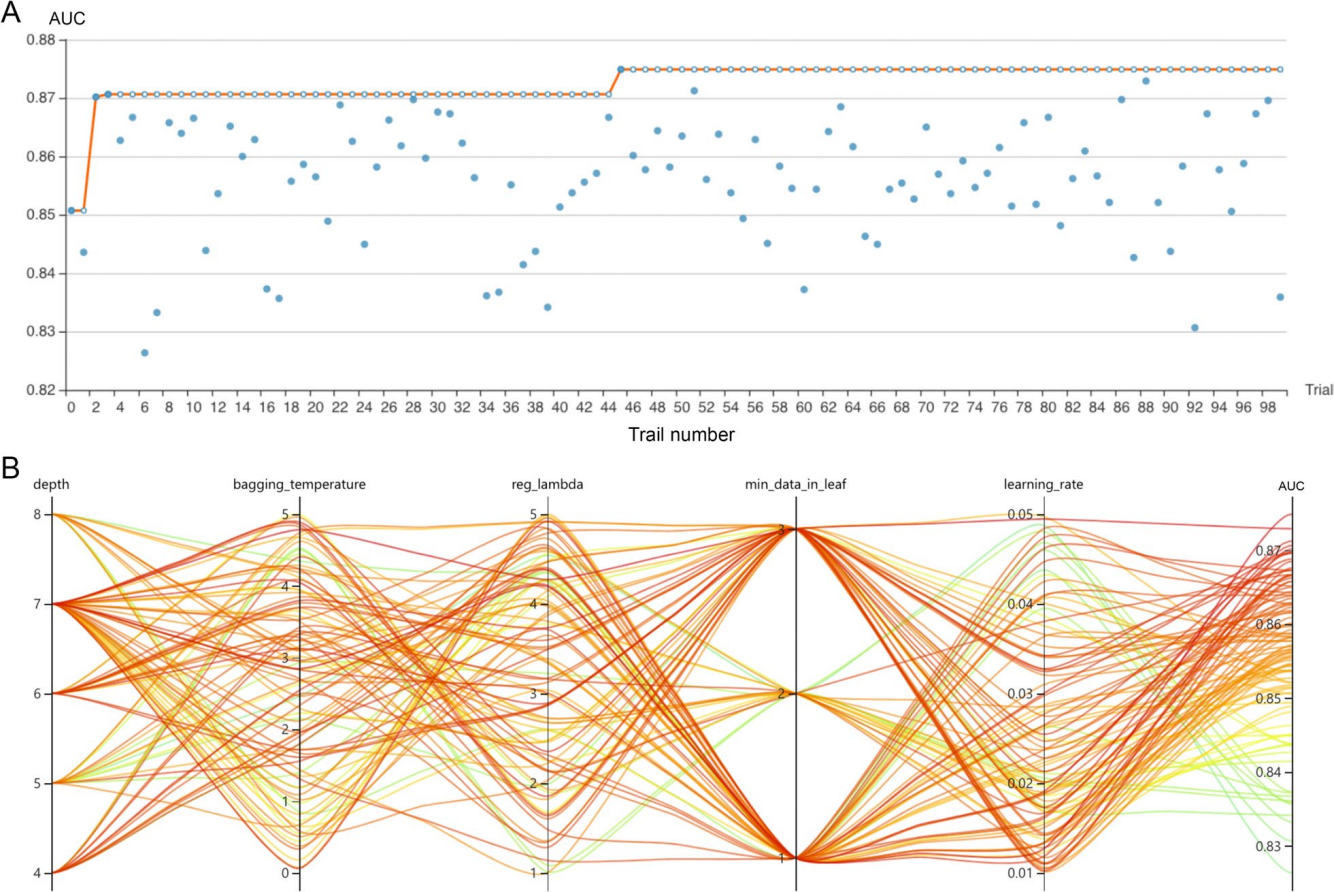
Hyperparameter optimization process with an automated machine learning toolkit. **A** The blue point represents the result of a trail, and the dark orange line represents the best area under the receiver operating characteristic curve (AUROC). **B** Each line represents a trail, and the green to red color line represents its AUROC

The RFE algorithm was implemented, and 93 NIV-failure-related indicators were initially screened (shown in Fig. 2A). Hyperparameter optimization was then conducted (shown in Fig. 2C). The top 15 key features were finally selected, including duration from extubation to NIV initiation (≤ 24 h/> 24 h), age, GCS, heart rate, respiratory rate, MBP, SpO_2_, temperature, glucose, pH, PaO_2_, urine output, input volume, mechanical ventilation duration, and mean airway pressure. Every variable was measured after NIV initiation within 6 h. After 100 trails, the CatBoost model with the greatest AUROC was obtained (shown in Fig. 3). The final settings of the hyperparameter search are listed in Additional file 2: Table S2.

**Fig. 4:**
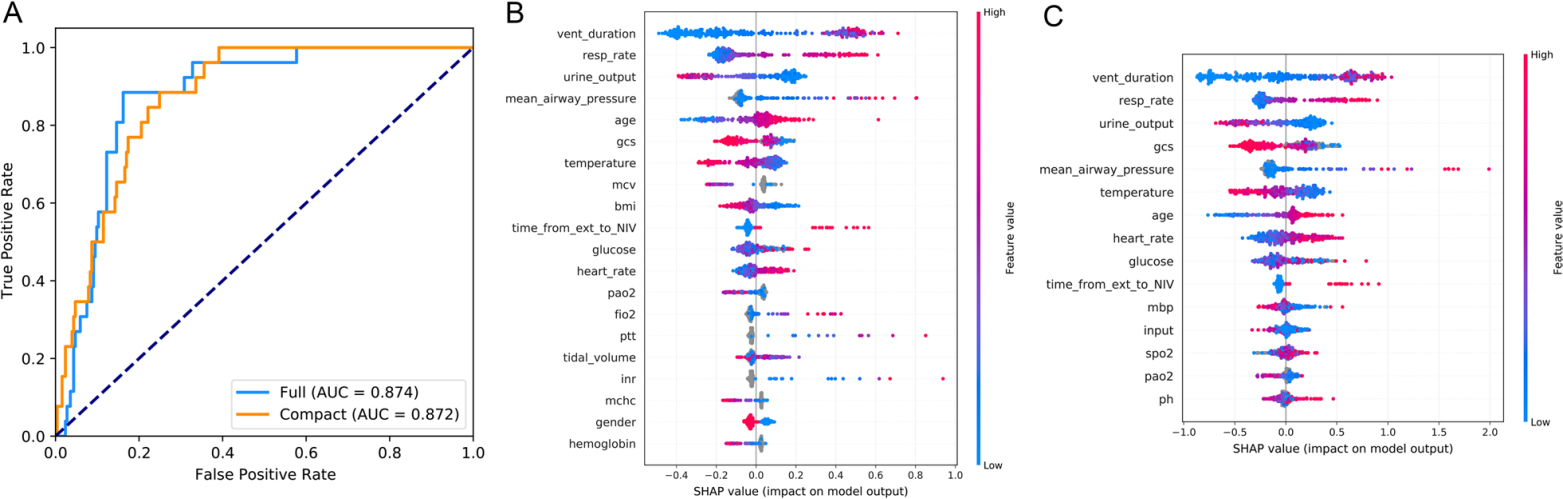
Comparison of the full and compact CatBoost models. The full model was developed on the basis of all available features, whereas the compact model was derived on the basis of key features selected by the recursive feature elimination algorithm. Both models had optimized hyperparameters. **A** Receiver operating characteristic curves (ROCs) of the full and the compact models. Distribution of the effects of each feature on the output of **B** the full model or **C** the compact model, estimated using the SHapley Additive exPlanations (SHAP) values. The plot sorts features by the sum of SHAP value magnitudes over all samples. The blue to red color represents the feature value (red high, blue low). The x-axis measures the effects on model output (right positive, left negative)

As shown in Fig. 4A, the full CatBoost model with 93 variables had an AUC of 0.874, whereas the compact CatBoost model with 15 key available variables had a remarkable AUROC of 0.872. Meanwhile in Fig. 4B–C, respectively, SHAP values for the two models were assessed on the internal validation set. Feature values were illustrated on a spectrum with blue representing the lowest value. The positive SHAP value indicates an increase in the risk of NIV failure and vice versa. According to the sum of absolute SHAP values over all samples, features were ranked [21]. As shown, respiratory rate, urine output, and mean airway pressure were also among the top important features. Furthermore, the MV duration was the most important variable for the prediction of NIV failure in the final model, and a longer duration indicated a higher NIV failure risk.

**Fig. 5:**
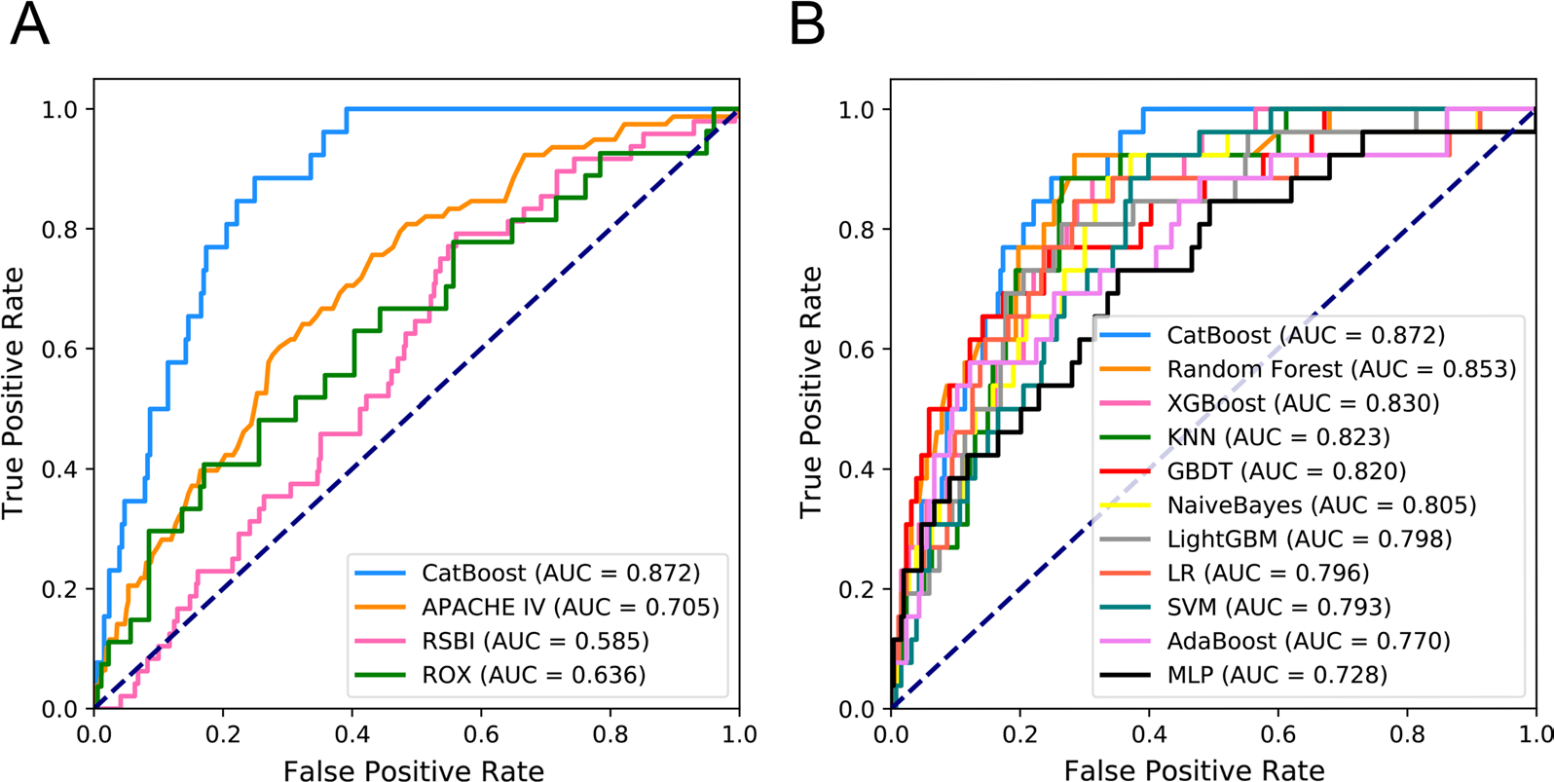
Comparison of model performance with other predictive tools and in the internal validation set. **A** Receiver operating characteristic curves (ROCs) of CatBoost and other predictive tools/factors. **B** Receiver operating characteristic curves (ROCs) of different models

Figure 5A, B depicts the comparison between the CatBoost model and other commonly used predictive factors or models. As shown, our CatBoost model significantly outperformed other predictive factors or models and had the greatest AUROC. Besides, we compared our model with HACOR score put forward by Duan et al. In view of different types of included patients between two studies(Duan’s study is for COPD patients vs ours is for extubated patients), we just listed the model results in the Additional file 3: Figure S1. For simplicity, only the results of CatBoost and LR are shown. The sensitivity and specificity analyses of these predictive methods on an internal validation set are summarized in Table 2. The sensitivity and specificity in the prediction group is 89% and 75%, while in the validation group they are 90% and 70%. The Youden Index for the two groups is 64% and 62% respectively. Decision Curve Analysis (DCA) is also listed in the Additional file 4: Figure S2. Although the CatBoost model did not have the best performance in all measures, it had the greatest Youden Index, which is considered a more comprehensive evaluation approach.

**Table 2:** Model performance in the internal and prospective validation sets

Model	AUC	ACC (%)	Best cutoff	Gray zone	Values in gray zone	Youden index (%)	Sensitivity (%)	Specificity (%)	F1 Score	PPV (%)	NPV (%)
*Internal validation*											
CatBoost	0.87 (0.82–0.92)	76 (71–81)	0.045	0.04–0.08	64 (22.94%)	64	89 (75–100)	75 (70–80)	0.41 (0.29–0.52)	27 (18–36)	98 (96–100)
Random Forest	0.85 (0.78–0.92)	73 (68–78)	0.077	0.08–0.14	51 (18.28%)	64	92 (80–100)	72 (66–77)	0.39 (0.27–0.50)	25 (16–34)	99 (97–100)
XGBoost	0.83 (0.76–0.89)	71 (66–76)	0.007	0.00–0.04	241 (86.38%)	57	89 (76–100)	69 (64–74)	0.36 (0.25–0.47)	23 (14–31)	98 (96–100)
KNN	0.82 (0.75–0.89)	75 (70–80)	0.05	0.04–0.09	85 (30.47%)	62	89 (74–100)	74 (68–79)	0.40 (0.28–0.50)	25 (17–35)	98 (96–100)
GBDT	0.82 (0.73–0.90)	76 (71–81)	0.03	0.01–0.06	161 (57.71%)	52	77 (60–92)	76 (70–81)	0.37 (0.25–0.48)	24 (15–34)	97 (94–99)
NaiveBayes	0.81 (0.72–0.88)	66 (61–71)	0.013	0.01–0.25	97 (34.77%)	55	93 (80–100)	63 (57–69)	0.33 (0.24–0.43)	20 (14–28)	99 (97–100)
LightGBM	0.80 (0.71–0.87)	74 (69–79)	0.004	0.00–0.03	242 (86.74%)	54	81 (64–95)	74 (68–79)	0.37 (0.25–0.48)	24 (15–33)	97 (95–99)
LR	0.80 (0.70–0.89)	73 (67–78)	0.055	0.02–0.12	155 (55.56%)	56	85 (69–97)	72 (66–77)	0.36 (0.25–0.47)	23 (15–32)	98 (96–99)
SVM	0.79 (0.72–0.86)	63 (57–68)	0.066	0.07–0.12	80 (28.67%)	52	93 (80–100)	60 (54–66)	0.32 (0.22–0.41)	19 (12–26)	99 (97–100)
AdaBoost	0.77 (0.66–0.86)	85 (81–89)	0.486	0.47–0.49	118 (42.29%)	45	58 (38–76)	88 (83–92)	0.42 (0.27–0.55)	33 (20–47)	95 (92–98)
COX	0.75 (0.64–0.84)	71 (66–76)	0.242	0.15–0.43	135 (48.39%)	47	77 (59–93)	70 (65–76)	0.32 (0.22–0.43)	21 (13–29)	97 (94–99)
MLP	0.73 (0.62–0.83)	66 (60–71)	0.001	0.00–0.04	241 (86.38%)	38	74 (55–91)	65 (59–71)	0.28 (0.18–0.38)	17 (11–25)	96 (93–99)
*Prospective validation*											
CatBoost	0.85 (0.80–0.89)	72 (68–77)	0.062	0.06–0.11	85 (20.29%)	62	92 (84–98)	70 (65–74)	0.44 (0.35–0.52)	29 (22–36)	98 (97–100)
XGBoost	0.81 (0.76–0.86)	67 (63–72)	0.014	0.01–0.15	149 (35.56%)	53	88 (78–96)	64 (60–69)	0.40 (0.31–0.47)	26 (19–32)	98 (95–99)
GBDT	0.81 (0.76–0.85)	66 (61–70)	0.047	0.04–0.18	150 (33.94%)	51	88 (78–96)	63 (58–68)	0.36 (0.28–0.44)	23 (17–29)	98 (96–99)
Random Forest	0.80 (0.75–0.85)	74 (70–78)	0.167	0.11–0.23	164 (37.10%)	50	76 (64–88)	74 (70–78)	0.40 (0.31–0.49)	27 (20–35)	96 (94–98)
COX	0.76 (0.70–0.81)	67 (62–71)	0.38	0.37–0.60	151 (34.16%)	52	88 (79–96)	64 (59–69)	0.37 (0.30–0.45)	24 (18–30)	98 (96–99)
LightGBM	0.74 (0.67–0.80)	68 (63–72)	0.013	0.00–0.09	358 (85.44%)	34	67 (54–80)	68 (63–72)	0.33 (0.25–0.41)	22 (16–29)	94 (91–97)
AdaBoost	0.72 (0.64–0.79)	67 (63–72)	0.483	0.47–0.49	275 (62.22%)	41	74 (61–86)	66 (62–71)	0.34 (0.26–0.42)	22 (16–29)	95 (92–98)
KNN	0.70 (0.63–0.77)	68 (64–72)	0.039	0.01–0.07	272 (61.54%)	31	62 (49–75)	69 (64–73)	0.30 (0.22–0.38)	20 (14–27)	93 (90–96)
LR	0.68 (0.60–0.76)	79 (75–83)	0.085	0.02–0.11	273 (61.76%)	34	52 (37–65)	82 (78–86)	0.36 (0.25–0.45)	27 (18–36)	93 (90–96)
NaiveBayes	0.67 (0.59–0.74)	65 (61–70)	0.021	0.00–0.15	387 (87.56%)	28	62 (50–76)	66 (61–70)	0.29 (0.21–0.36)	19 (14–25)	93 (90–96)
MLP	0.66 (0.58–0.74)	58 (54–63)	0.005	0.00–0.27	391 (88.46%)	32	76 (64–88)	56 (51–61)	0.29 (0.22–0.36)	18 (13–23)	95 (92–98)
SVM	0.65 (0.58–0.72)	51 (46–55)	0.055	0.04–0.14	305 (69.00%)	32	86 (75–95)	46 (41–51)	0.28 (0.21–0.35)	17 (12–22)	96 (93–99)

Models are ordered according to the area under the receiver operating characteristic curve. The Youden index was defined as sensitivity + specificity − 1.

XGBOOST, eXtremely Gradient Boosting; GBDT, Gradient Boosting Decision Tree; KNN, K-Nearest Neighbor; SVM, Support Vector Machine; MLP, Multi-Layer Perceptron; LR, Logistic Regression; PPV, positive predictive value; NPV, negative predictive value

### Prospective validation and a web-based tool

**Fig. 6:**
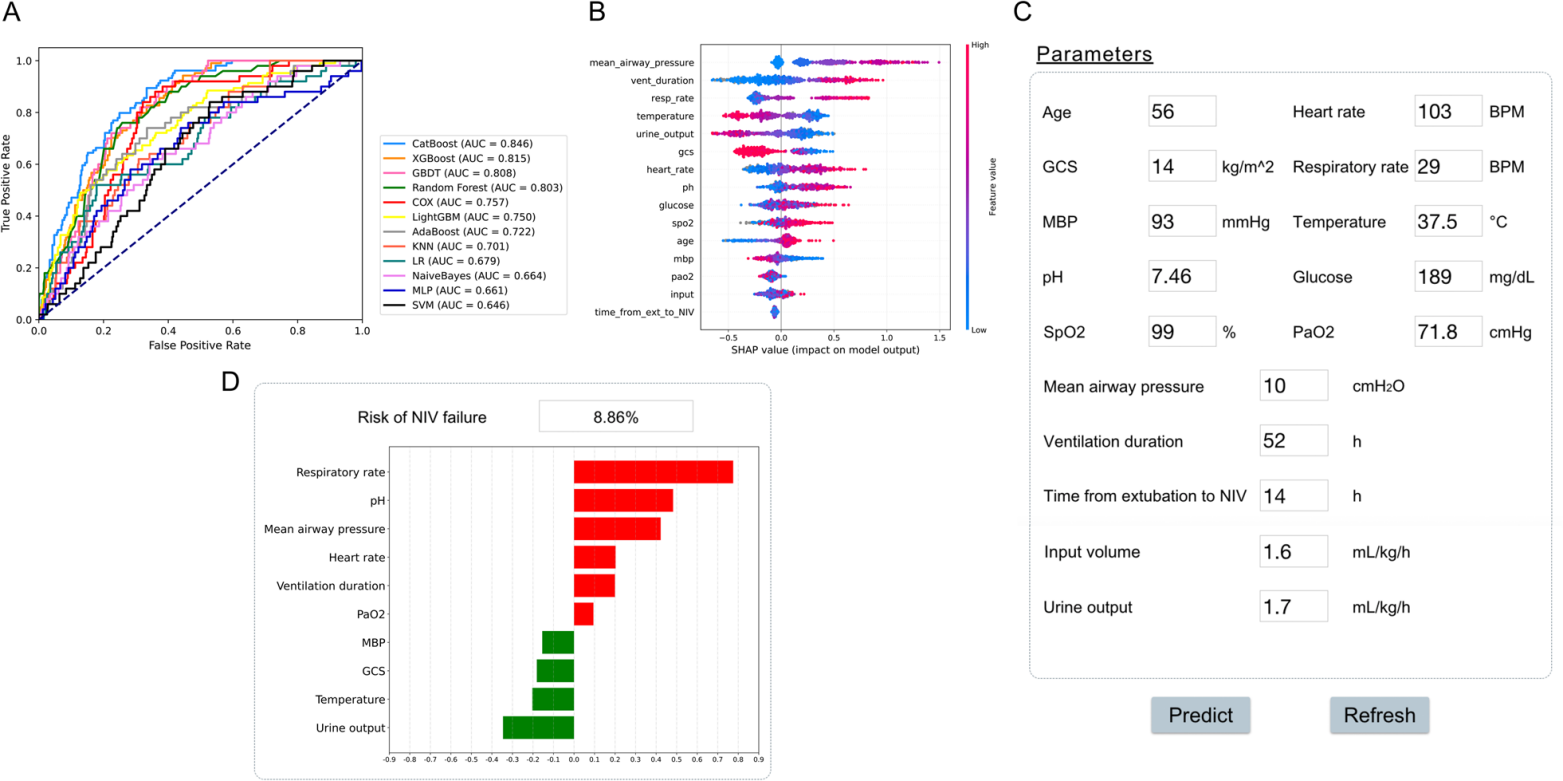
Application of the CatBoost model. **A** Receiver operating characteristic curves of different models in the prospective validation set. **B** Influence of the SHAP value on model output. **C** An example of the web-based tool. **D** The prediction results of CatBoost model and the top ten importance features are summarized. A green bar indicates a protective factor, whereas a red bar represents a risk factor. Bar length corresponds to the magnitude of protection or risk

We additionally assessed the ability of our final model to discriminate patients after extubation who were unlikely to benefit from NIV according to the CatBoost model. The results of prospective validation are shown in Fig. 6A, B. Our model also had a better generalization ability (AUROC: 0.846 [95% CI 0.82–0.92]) than the other nine models. The sensitivity and specificity analysis are also summarized in Table 2.

On the basis of the final machine-learning model, a web-based tool was also established to allow clinicians to use the compact model: http://www.aimedicallab.com/tool/aiml-nivfailure.html. An example of using our tool is depicted in Fig. 6C. Users enter the variable values when the patient receives NIV within 6 h, leaving missing values blank, then click the ‘predict’ button. The risk of NIV failure is assessed by the CatBoost model, and the top 15 important features are returned, as shown in Fig. 6B.

## Discussion

In this study, we developed and validated a predictive machine-learning model for extubated critically ill patients receiving NIV within 6 h. This model, based on 15 key parameters (mechanical ventilation duration, RR, urine output, GCS, mean airway pressure, temperature, age, heart rate, glucose, time from extubation to NIV, mean blood pressure, input volume, SpO_2_, PaO_2_, and pH) in the first 6 h of NIV, had a discriminatory ability of 0.872 (AUROC value of compact model) and an AUROC of 0.846 in the PREDICt cohort. To our knowledge, this is the first model constructed on a large-scale public database and then further prospectively validated in a university teaching hospital to early predict NIV failure after extubation. In addition, it is the first model to predict NIV failure with machine learning. Moreover, in contrast to previously published models, we provide an open and accessible data interface for the public to use and validate our model. The extubated patients needing NIV within 48 h, unlike those with successful extubation, were in more serious condition, and had poorer prognosis and a higher risk of death. To avoid delayed reintubation, timely use of our model and accurately predicting NIV failure within 6 h could help clinicians identify high-risk patients and adjust further treatment decisions.

The SHAP value chart may reveal the indicators on which attention should be focused. SHAP values can reveal the importance of each feature through a game-theory approach. Feature values were indicated by a spectrum, with blue representing the lowest value. A positive SHAP value represents an increase in the risk of NIV failure, whereas a lower value indicates decreased risk. Features were ranked according to the sum of absolute SHAP values over all samples. By assessing SHAP values, we found that MV duration and respiratory rate were the most important features for prediction, in agreement with prior studies. Furthermore, urine output, GCS, and mean airway pressure were also shown to be associated with NIV failure. In addition, the NIV failure rate in the PREDICt cohort was approximately 26%, thus confirming the results of previous reported studies. We further verified the previous predictive scale reported by Duan et al. for NIV failure in selected patients with COPD (based on heart rate, acidosis, consciousness, oxygenation, and respiratory rate) with data including the above parameters in the eICU-CRD group. Our machine learning model Catboost also includes these indicators and we found that it has an AUC of 0.872. Our study aims at the prediction of NIV failure among a more general population after extubation, confirming that these above parameters play a significant role.

The timing of initiation of NIV treatment has been always a crucial aspect. Among patients whose duration from extubation to NIV was 24 h or less in the PREDICt cohort (n = 876), approximately 26% (n = 229) experienced NIV failure. In the validation cohort, it is about 17% (n = 59). This result may indicate the potential positive effect of early initiation of NIV after extubation. It may be consistent with those from previous studies showing that early use NIV is associated with a significant reduction of post-extubation respiratory failure [19, 25]. However, this finding must be validated in further prospective studies [14–18, 21].

To make this model easier to apply at bedside, we artificially removed some of the more complex ratings. Initially, 93 variables were evaluated, and we eventually selected 15 key features that could be easily obtained, thus, improving the model’s usability and convenience over those in previous studies. As expected, the slight decrease in the AUROC of the compact model based on selected features (Fig. 4A) demonstrated that other variables could be excluded without substantially decreasing model performance. The efficacy of these features on the prediction results were then assessed with SHAP values. Although SAPS-II, APACHE-II, and other risk scores have been shown to be important for prediction in previous studies as well as in our study, we still did not incorporate these features to ensure relatively high accuracy. The main reason for this decision was that those scores greatly increase the computational complexity, and would make our model inconvenient to use in clinical settings [11]. In terms of the GCS, clinicians must assess patient consciousness, particularly in patients with conditions such as COPD or hypercapnia who receive NIV. This score can also be evaluated quickly and completely at the bedside, and therefore, remained in the rating scale.

The CatBoost model derived with optimized hyperparameters, on the basis of the key features described above, outperformed other predictive factors and nine models on the eICU-CRD dataset. However, CatBoost, as a gradient boosting algorithm, has not yet been widely adopted in critical care research, although prior studies have reported that CatBoost significantly outperforms other machine-learning models in various tasks. Besides, CatBoost can successfully accommodate categorical features and some missing values automatically, and advantageously handles them during training instead of preprocessing time. Therefore, missing values do not need to be imputed and categorical features no longer need to be encoded. On the other hand, the algorithm uses a new schema to calculate leaf values when selecting the tree structure. It may help reduce overfitting, the major problem constraining the generalization ability of machine-learning models [12].

Our model is beneficial for clinical and medical resources as well as economic savings. In addition to external validation, to prospectively validate our model, more than 400 patients were enrolled from the CSICU of Zhongshan Hospital, Fudan University. Our model showed a greater AUROC (in Fig. 6) than other models, indicating its remarkable generalization ability and clinical value. And we developed a web-based tool to help clinicians use the model, which provides a user-friendly interface [12]. After entering the variable values, the tool returns the risk of NIV failure as well as the top 15 important features. These results should help clinical decision-makers understand patient status and pursue appropriate treatment strategies. More importantly, our model is a promising tool to improve the prognosis of patients undergoing NIV, and may have extremely positive effects medically.

Several important limitations of this study must be considered. First, some novel parameters or techniques proposed by recent studies were not included in our study, such as central venous-to-arterial P_CO2_ difference [26], thenar oxygen saturation [27], and diaphragm dysfunction [28]. We argue that these parameters or techniques require multiple measurements or complex calculations, thus hindering application in clinical settings. The variables selected in our study can be rapidly determined and directly measured, thus improving the model’s practicality. Second, the sensitivity and specificity of our model were 89% and 75%, respectively; therefore, the false positive rate may be relatively high. Several patients might be incorrectly predicted to have failure and thus unnecessarily consume medical resources. Third, the parameter monitoring in NIV may not be accurate. For example, the included RR and mean airway pressure may lead to errors in the parameters due to patient intolerance during NIV, patient-ventilator asynchronies and other reasons; therefore, close monitoring and observation by medical staff are needed. Finally, because the PREDICt cohort was performed in the CS-ICU, the types of patients enrolled in the validation study are limited, and consequently, the efficacy of this model might be skewed. In the future, a more diverse group of patients and randomized controlled trials will be needed to further verify the diagnostic power.

### Implications

As shown in previous studies, NIV failure is independently associated with higher mortality. Reintubation is also accompanied by the occurrence of complications such as acute respiratory distress syndrome, sepsis, ventilator-associated pneumonia, prolonged ICU stays, and increased medical costs. With this model, if a patient is predicted to have a high risk of NIV failure, more intensive monitoring could be provided, and/or earlier intubation might be considered, thus potentially reducing mortality. The model’s clinical value will be further assessed and reported in future prospective studies.

## Conclusions

In conclusion, the present study screened 15 key features associated with NIV failure in patients whose NIV was initiated within 6 h from extubation, and developed a CatBoost model that outperforms existing models in predicting NIV failure, particularly early NIV failure within 6 h from attempted extubation. Because the machine-learning model is based on variables easily determined at bedside, it can be conveniently used to assess the efficacy of NIV in general populations after extubation. In higher risk patients, early intubation or other promising therapies should be considered by clinicians.

## Supplementary Information

**Additional file 1: Table S1.** Features extracted from the eICU-CRD database and the key features selected

**Additional file 2: Table S2.** Hyperparameter search domains and final settings

**Additional file 3: Figure S1.** Comparison of model performance in eICU Collaborative Research Database set. (A) Receiver operating characteristic curves (ROCs) of CatBoost and other predictive factors. (B) Receiver operating characteristic curves (ROCs) of different models.

**Additional file 4: Figure S2.** Decision Curve Analysis on CatBoost and Logistic Regression

## Data Availability

The datasets used and/or analyzed during the current study are available from the corresponding author.

## Abbreviations

NIV: Noninvasive ventilation
IMV: Invasive mechanical ventilation
eICU-CRD: EICU Collaborative Research Database
CatBoost: Categorical Boosting
GCS: Glasgow Coma Scale score
MBP: Mean blood pressure
SpO_2_: Saturation of pulse oxygen
PaO_2_: Partial arterial oxygen pressure
TRIPOD: Transparent Reporting of a multivariable prediction model for Individual Prognosis or Diagnosis
RFE: The recursive feature elimination algorithm
SHAP: SHapley Additive exPlanations
SOFA: Sequential Organ Failure Assessment
SAPS-II: Simplified Acute Physiology Score

## Footnotes

1 Reporting Checklist: The authors have completed the TRIPOD reporting checklist. All authors have completed the ICMJE uniform disclosure form. The authors have no conflicts of interest to declare.

2 The authors are accountable for all aspects of the work in ensuring that questions related to the accuracy or integrity of any part of the work are appropriately investigated and resolved.
