# Supplementary material for: Early prediction of noninvasive ventilation failure after extubation: development and validation of a machine-learning model
Source: BMC Pulm Med. 2022 Aug 8;22:304. doi: 10.1186/s12890-022-02096-7 (PMC9358918; doi:10.1186/s12890-022-02096-7)
Supplement: Supplementary file 2 — Additional file 2: Table S2. Hyperparameter search domains and final settings [file 12890_2022_2096_MOESM2_ESM.docx]

Table S2. Hyperparameter search domains and final settings

| Hyperparameters | Type | Search domain | Final setting |
| --- | --- | --- | --- |
| 'depth' | Choice | {4, 5, 6, 7, 8} | 4 |
| 'bagging_temperature' | Uniform | [0, 5] | 2.23 |
| 'reg_lambda' | Uniform | [1, 5] | 1.98 |
| 'learning_rate' | Uniform | [0.01, 0.05] | 0.039 |
| 'min_data_in_leaf' | Choice | {1, 2, 3} | 1 |
