# Supplementary figures and images for: Early prediction of noninvasive ventilation failure after extubation: development and validation of a machine-learning model
Source: BMC Pulm Med. 2022 Aug 8;22:304. doi: 10.1186/s12890-022-02096-7 (PMC9358918; doi:10.1186/s12890-022-02096-7)

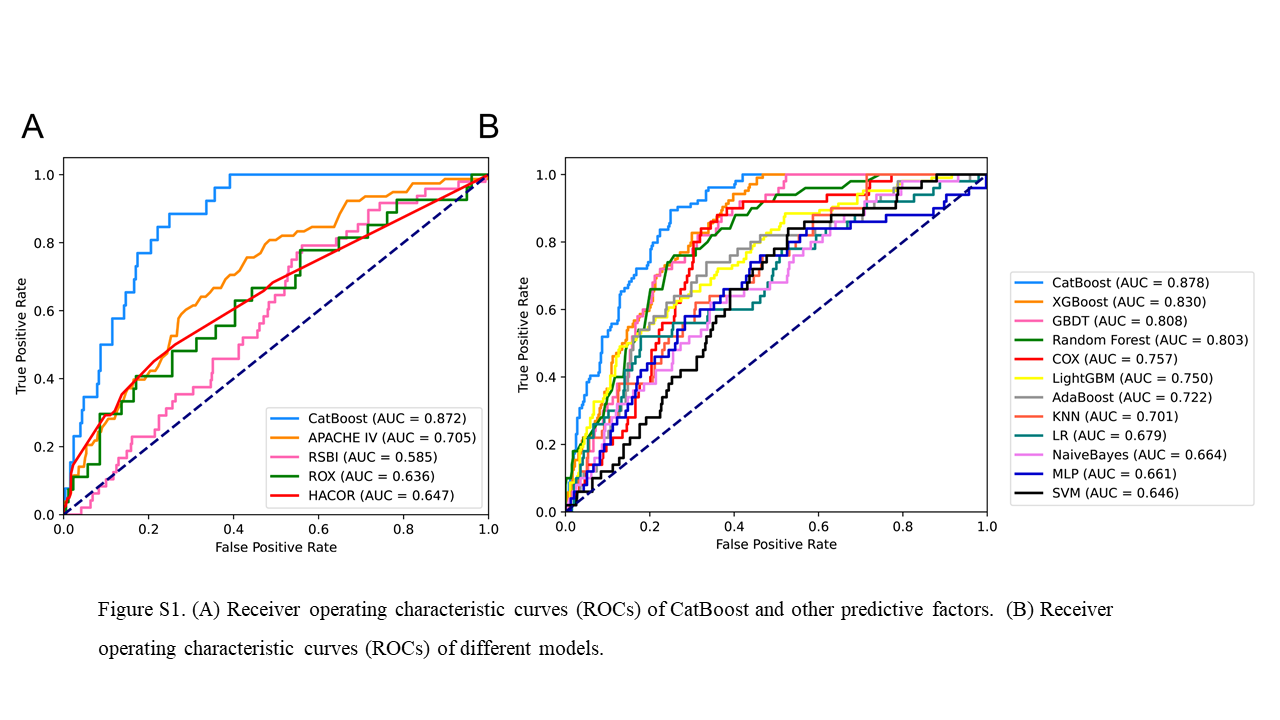

Supplement: Supplementary file 3 — Additional file 3: Figure S1. Comparison of model performance in eICU Collaborative Research Database set. (A) Receiver operating characteristic curves (ROCs) of CatBoost and other predictive factors. (B) Receiver operating characteristic curves (ROCs) of different models. [file 12890_2022_2096_MOESM3_ESM.tif]

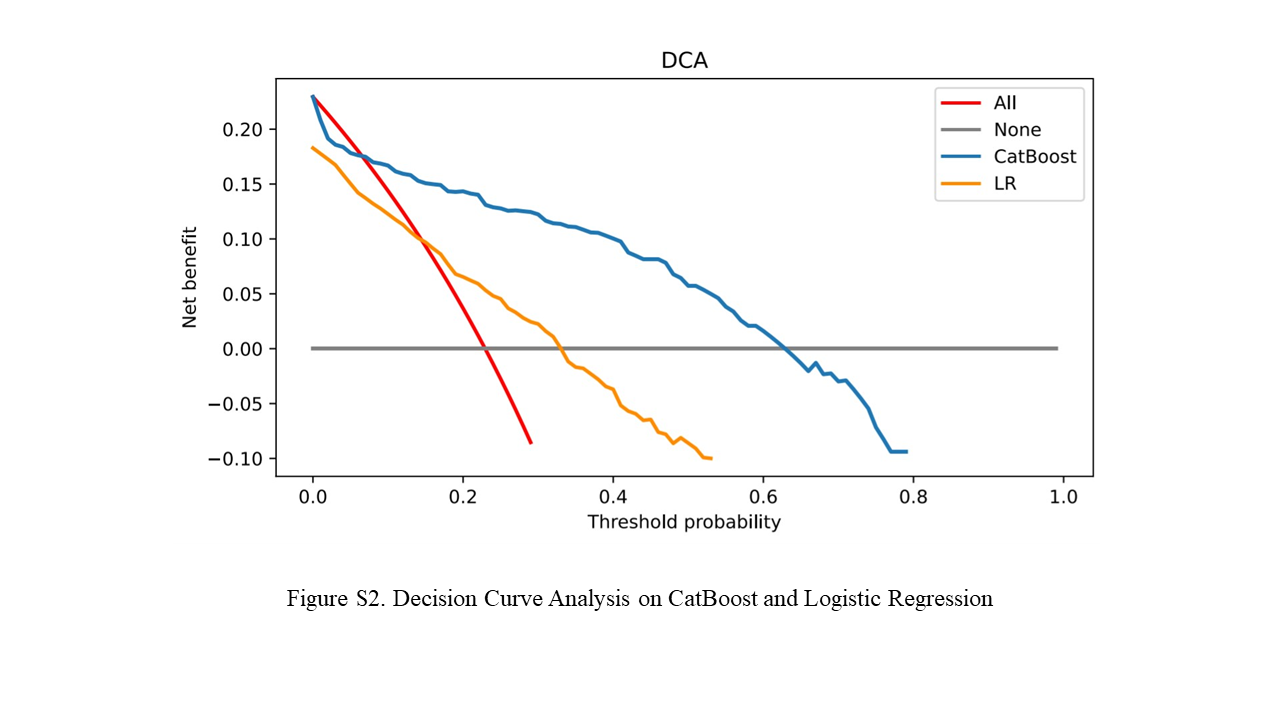

Supplement: Supplementary file 4 — Additional file 4: Figure S2. Decision Curve Analysis on CatBoost and Logistic Regression [file 12890_2022_2096_MOESM4_ESM.tif]
